# Heparin-Coated Dendronized Hyperbranched Polymers for Antimalarial Targeted Delivery

**DOI:** 10.1021/acsapm.2c01553

**Published:** 2022-12-30

**Authors:** María San Anselmo, Elena Lantero, Yunuen Avalos-Padilla, Inés Bouzón-Arnáiz, Miriam Ramírez, Alejandro Postigo, José Luis Serrano, Teresa Sierra, Silvia Hernández-Ainsa, Xavier Fernàndez-Busquets

**Affiliations:** † Instituto de Nanociencia y Materiales de Aragón (INMA), Departamento de Química Orgánica-Facultad de Ciencias, CSIC-Universidad de Zaragoza, Zaragoza 50009, Spain; ‡ Nanomalaria Group, Institute for Bioengineering of Catalonia (IBEC), The Barcelona Institute of Science and Technology, Baldiri Reixac 10-12, Barcelona 08028, Spain; § Barcelona Institute for Global Health (ISGlobal, Hospital Clínic-Universitat de Barcelona), Rosselló 149-153, Barcelona 08036, Spain; ∥ Nanoscience and Nanotechnology Institute (IN2UB), University of Barcelona, Martí I Franquès 1, Barcelona 08028, Spain; ⊥ ARAID Foundation, Government of Aragón, Zaragoza 50018, Spain

**Keywords:** dendritic polymers, targeted drug delivery, malaria, nanocarriers, heparin

## Abstract

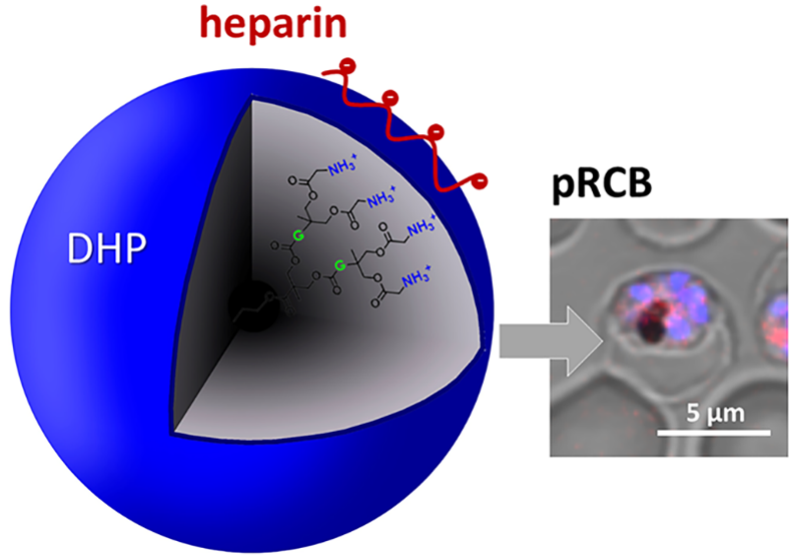

The rampant evolution of resistance in *Plasmodium* to all existing antimalarial drugs calls for the development of improved therapeutic compounds and of adequate targeted delivery strategies for them. Loading antimalarials in nanocarriers specifically targeted to the parasite will contribute to the administration of lower overall doses, with reduced side effects for the patient, and of higher local amounts to parasitized cells for an increased lethality toward the pathogen. Here, we report the development of dendronized hyperbranched polymers (DHPs), with capacity for antimalarial loading, that are coated with heparin for their specific targeting to red blood cells parasitized by *Plasmodium falciparum*. The resulting DHP–heparin complexes exhibit the intrinsic antimalarial activity of heparin, with an IC50 of *ca.* 400 nM, added to its specific targeting to *P. falciparum*-infected (*vs* noninfected) erythrocytes. DHP–heparin nanocarriers represent a potentially interesting contribution to the limited family of structures described so far for the loading and targeted delivery of current and future antimalarial compounds.

## 1 Introduction

In the search for antimalarial strategies oriented to make a better use of the few available drugs and of potential future compounds to be discovered,^1^ encapsulation in targeted nanocarriers offers several advantages.^2^ The usually large biodistribution of antimalarials in the organism requires the administration of high doses to account for the losses resulting from drug intake by cells and tissues other than the main target cell in clinical malaria, the *Plasmodium*-infected red blood cell (RBC). Remaining below overall toxic drug levels for the patient usually leads to sublethal local amounts that are the ideal scenario for resistance evolution. Many antimalarial drugs have shown good therapeutic efficacy, but there is still room for improvement in avoiding side effects, reducing toxicity, increasing half-life, ameliorating bioavailability, preventing rapid drug resistance emergence, and decreasing the dosage for an effective treatment. Nanotechnology-based drug delivery systems are novel tools well placed to improve the efficacy of current antimalarial drugs and overcome their limitations. Nanocarriers can be designed to target specific molecules, protect the drug from degradation, prolong blood circulation time, cut down dose frequency, overcome side effects, improve the pharmacokinetic profile, and encapsulate several drugs in the same nanostructure, which can significantly boost treatment efficacy.^3−6^

As a targeting element capable of substituting for antibodies, the natural polysaccharide heparin had promising perspectives especially regarding liposome delivery to *Plasmodium* late blood stages.^7,8^ Heparin offered two additional benefits, namely, its antimalarial activity^7,9−11^ and its targeting to ookinetes,^12,13^ the motile mature zygote in the midgut of the mosquito species of the *Anopheles* genus that transmit malaria, which paved the way for future antimalarial strategies blocking the parasite’s life cycle in the mosquito vector.^14^ Heparin has been used in the past for the treatment of severe malaria,^15−17^ but it was abandoned because of its strong anticoagulant action, with side effects such as intracranial bleeding.^18^ However, the formation of polyelectrolyte stable complexes between macromolecular structures such as polymers and heparin has been explored as a strategy to reduce its hemorrhagic activity.^19^ These results suggested that the use of heparin as a targeting element of polymeric nanocarriers could provide a new generation of versatile, cost-efficient nanomedicines against malaria parasites.

Because eventual antimalarial nanomedicines need to be deployed in low per capita income regions, their final components must take into account this particular economic landscape. Whereas the results provided by liposomes as nanocapsules and antibodies as targeting elements of antimalarial drug-loaded nanocarriers offered good performance *in vitro* and *in vivo*,^20−22^ their production cost was too high for widespread use in malaria endemic regions. This spurred the development of more affordable technologies, which initially materialized with the development of polymeric nanocarriers as encapsulating structures^23−26^ and heparin as the targeting molecule. Indeed, according to our own calculations,^12^ since heparin is significantly less expensive to obtain than specific (monoclonal) antibodies, heparin-targeted antimalarial nanocarriers were estimated to be about 10 times less expensive than equally performing immunoliposomes. Similarly, preliminary rough estimations for such heparin-nanoparticle systems indicate that the cost of synthesizing polymeric nanocarriers would be at least 1 order of magnitude lower than that of a liposomal system having an equal antiplasmodial *in vitro* activity. Therefore, heparin-dendrimer nanocarriers would be at least 100 times less costly to produce than their liposomal counterparts. In addition, we must consider that polymers are much more adaptable than liposomes to oral administration, which is the optimal delivery route for antimalarial therapeutics and chemoprophylaxis.

**Figure 1:**
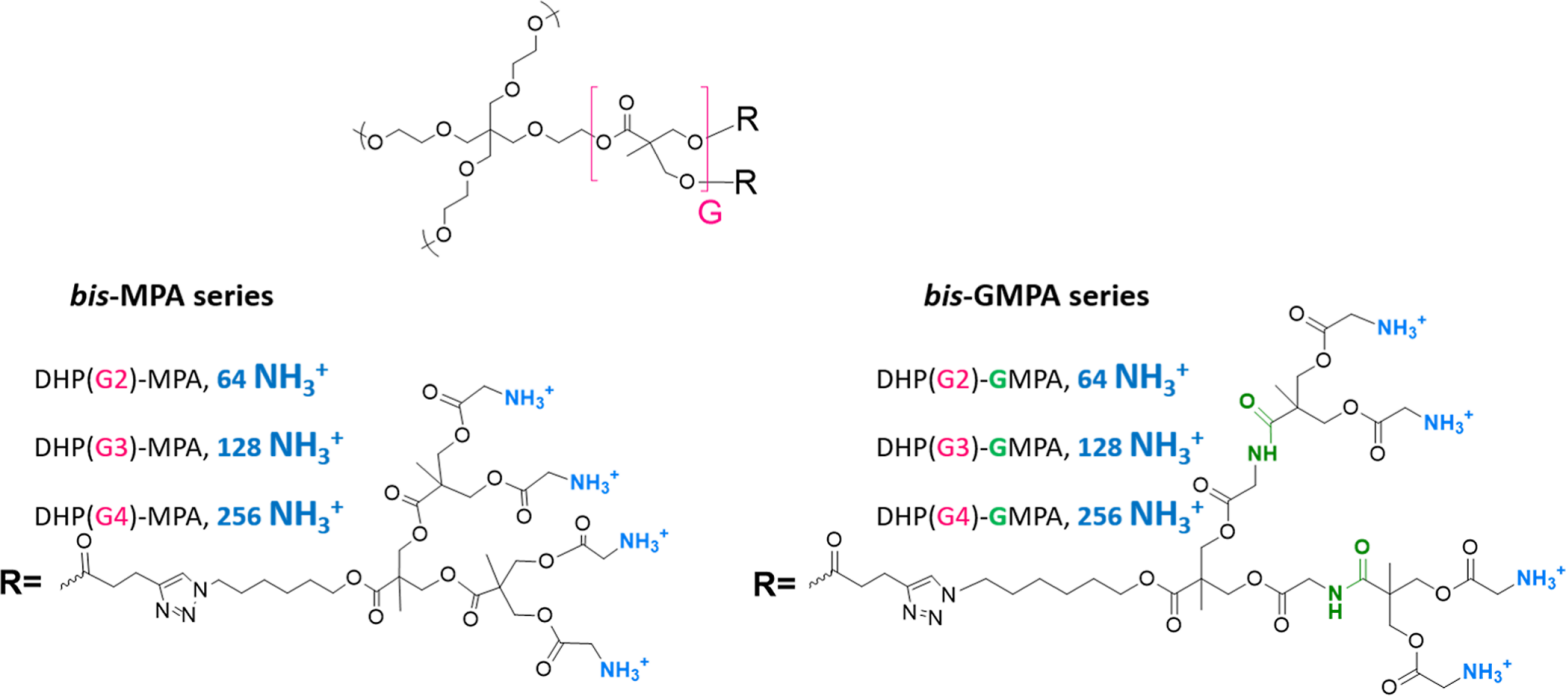
Chemical structures of the investigated DHPs.

Among different polymeric materials, dendrimers count with branched structures and globular shapes constituting unique features that support their use as nanocarriers for drug delivery. In fact, these characteristics help to decrease renal filtration and increase blood circulation times thus favoring their diffusion to target cells and the efficiency of the therapeutic effect.^27^ Furthermore, current intense attention is directed at analyzing the *in vitro*(28) and *in vivo*(29) physicochemical parameters that make dendrimers nanomaterials for drug delivery with appropriate pharmacokinetic/pharmacodynamic properties for different diseases, including malaria.^30,31^ In this respect, our group has described dendritic polymers consisting in Janus dendrimers, hybrid dendritic-linear-dendritic block copolymers, and dendronized hyperbranched polymers (DHPs) bearing architectures based on 2,2-*bis*(hydroxymethyl)propionic acid (*bis*-MPA) dendrons, which have shown interesting capabilities as nanocarriers for different antimalarial drugs (chloroquine, primaquine, and quinacrine) and targeted delivery toward parasitized RBCs (pRBCs).^25,32^ These good properties led us to investigate their potential to enhance the antimalarial and targeting characteristics of heparin. In particular, we propose here a series of heparin-dendritic nanocarriers based on DHPs of a *bis*-MPA core of three generations (G2, G3, and G4) bearing either *bis*-MPA or *bis*-GMPA [2,2′-*bis*(glycyloxymethyl) propionic acid] dendrons of generation 2. Namely, the *bis*-MPA series is composed by DHP(G2)-MPA, DHP(G3)-MPA, and DHP(G4)-MPA and the *bis*-GMPA series consists of DHP(G2)-GMPA, DHP(G3)-GMPA, and DHP(G4)-GMPA (Figure 1).

The cationic nature of these DHPs, arising from their peripheral ammonium groups, is exploited to obtain a strong electrostatic interaction with the negative charges in heparin. Previous results with one *bis*-MPA-derived DHP showed the potential of this conjugating strategy.^25^ The inclusion of the bis-GMPA architecture was thought to possibly modulate the characteristics of the obtained nanocarriers through interactions such as the hydrogen bonds mediated by amide groups. Consequently, we expect that the presence of either the *bis*-MPA or the *bis*-GMPA structure can exert an influence in the capacity of these DHPs to complex heparin, their targeting, and their antimalarial efficacy. DHPs of both series fluorescently labeled by functionalization with rhodamine B are also investigated.

## 2 Materials and Methods

### 2.1 Preparation of DHPs

Reagents were purchased from Sigma-Aldrich Corporation (St. Louis, MO, US) or Acros Organics, and the solvents were purchased from Fisher Scientific or Scharlab.

The synthesis and characterization of DHPs have been reported before.^33^ In brief, their synthesis involves the functionalization of commercial hyperbranched polyesters with an alkyne terminal group by Steglich esterification that is subsequently used to anchor the *bis*-MPA or *bis*-GMPA dendron (t-Boc protected) through a 1,3-dipolar cycloaddition click chemistry reaction and a final deprotection under acidic conditions. Rhodamine B (Rh)-labeled DHPs (DHPs-Rh) were prepared by covalent coupling of Rh with around 1% of the amino terminal groups of each DHP *via* amide formation. Characterization was done by ^1^H nuclear magnetic resonance (^1^H NMR) and ^13^C NMR, Fourier transform infrared spectroscopy, and size-exclusion chromatography.

### 2.2 Transmission Electron Microscopy

A droplet (10 μL) of a freshly prepared 1 mg/mL sample was deposited on a Formvar (10 nm)/carbon film (1 nm)-coated 400 mesh copper grids. Negative staining was performed with a 1% uranyl acetate solution, and transmission electron microscopy (TEM) images were recorded with a TECNAI T20 electron microscope (FEI Company, Hillsboro, OR, US) using a beam power of 200 kV.

### 2.3 Methylene Blue Competition Assay

The formation of complexes between the negatively charged glycosaminoglycan heparin (Bioiberica S.A.U., Palafolls, Spain; *ca.* 13 kDa mean molecular weight) and the polycationic DHPs (hereafter referred to as DHP/hep complex) was studied by the methylene blue (MB) UV–visible spectroscopic competition assay, adapted from Rodrigo *et al.*(34) and Al-Jamal *et al.*(35) Briefly, free MB has a maximum absorbance at 665 nm, which shifts to 565 nm upon association with heparin (see Figure S1), whereby the A665/A565 ratio indicates the fraction of free MB. In the competition assay designed, a fixed amount of heparin and the corresponding amount of MB that fully complexed the polysaccharide were previously established before incorporating the DHP, which competes with MB for heparin binding. The corresponding displacement can be observed by the appearance of a peak at 665 nm, which indicates that MB has been released from the complex and remains free in solution. Thus, the amount of free MB determined directly correlates with the quantity of heparin complexed by the DHP.

Initial complexes between heparin and MB (final respective concentrations of 10 μg/mL and 50 μM) were formed in 96-multiwell dishes in tris–HCl 10 mM, pH 7.4, under vigorous stirring at room temperature (RT) for 15 min. Then, increasing amounts of the corresponding DHP calculated as the weight ratio (*w*_DHP_/*w*_heparin_) were added and the final volume was adjusted to 150 μL with double-deionized water (ddH_2_O; Milli-Q system, Millipore Corporation, Burlington, MA, US). Ratios tested were 0.25, 0.5, 1, 1.5, 2, 3, 4, 5, 7.5, 10, 15, 20, 25, 30, and 40. The mixtures were allowed to stir for another 30 min at RT, and a spectroscopic scanning between 400 and 800 nm was performed in an Infinite M Nano+ instrument (Tecan Trading AG, Männedorf, Switzerland). Triplicates of all experiments were performed with the six DHPs of the *bis*-MPA and *bis*-GMPA series and with the homologues containing Rh.

### 2.4 Cytotoxicity Assays

The cytotoxicity of the series of DHPs and their labeled homologues was evaluated in human umbilical vein endothelial cells (HUVECs; American Type Culture Collection, Manassas, VA, US) by adapting pre-established protocols.^25^ Cells were grown in Medium 199 (M199, LabClinics, Barcelona, Spain) supplemented with 10% heat-inactivated fetal bovine serum (FBS; Invitrogen, US), 1% penicillin/streptomycin, and 10 mM glutamine, seeded at a density of 2.2 × 10^4^ cells/mL in MW96 plates and incubated for 24 h at 37 °C. Then, the medium was replaced with samples diluted in M199 without complements and plates were incubated for a further 48 h at 37 °C. The medium was then replaced by 10% 4-[3-(4-iodophenyl)-2-(4-nitrophenyl)-2*H*-5-tetrazolio]-1,3-benzene disulfonate (WST-1, Roche, Penzberg, Germany) in M199 and incubated under the same conditions for 4 additional hours. The cleavage of the WST-1 tetrazolium salt to yield formazan, occurring inside living cells, was spectrophotometrically determined by measuring absorbance at 440 and 600 nm in an EPOCH plate reader (BioTek, Agilent Technologies, Santa Clara, CA, US). Cells in the medium without any treatment were included as the positive growth control, cells in 50% dimethyl sulfoxide were used as the negative control, and the maximum percentage of water added with the samples (5% v/v) was also tested. Samples were assayed in triplicate.

### 2.5 Targeting Assays

*Plasmodium falciparum*-infected RBC cultures were synchronized at late stages by treatment in 70% Percoll (GE Healthcare, Uppsala, Sweden) density centrifugation at 1070×*g* for 10 min. Then, 2.5 mg/mL of either DHP(G4)-MPA/hep or DHP(G4)-MPA-Rh/hep was incubated with the synchronized pRBCs for 30 min at 37 °C. After this time, samples were washed twice with phosphate-buffered saline (PBS) and nuclei were counterstained with 2 μg/mL Hoechst 33342. For confocal microscopy analysis, samples were placed in a μ-Slide eight-well chamber slide (ibidi GmbH, Gräfelfing, Germany), and images were collected with a Leica TCS SP5 confocal fluorescence microscope (Mannheim, Germany) using a 63× oil immersion objective. Rhodamine was excited with a diode-pumped solid-state laser at 561 nm and Hoechst 33342 with a diode laser at 405 nm, and the fluorescence signal was collected in the range of 580–650 and 415–460 nm, respectively. To avoid crosstalk between the different fluorescence signals, sequential scanning was performed.

Flow cytometry targeting analysis was done in an LSRFortessa cytometer (BD Biosciences, San Jose, CA, US) set up with the five lasers, 20-parameter standard configuration. Hoechst 33342 and Rh signals were detected, respectively, by excitation with 350 nm/60 mW and 561 nm/100 mW lasers and emission collection with 450/50BP and 610/620BP nm bandpass filters.

### 2.6 *P. falciparumIn Vitro* Growth Inhibition Assays

*P. falciparum* parasites of the 3D7 strain were 5% sorbitol-synchronized as described elsewhere,^36^ in order to obtain a culture enriched in ring-stage parasites. After the synchronization process, a new culture at 1.5% parasitemia and 6% hematocrit was established and transferred to 96-well plates (Thermo Fisher Scientific, Waltham, MA, US). DHPs/hep and DHPs-Rh/hep were dissolved in the minimal amount of water and incubated for 30 min at RT before making serial dilutions in Roswell Park Memorial Institute 1640 medium (RPMI, Gibco, Thermo Fisher Scientific) containing l-glutamine and sodium bicarbonate and supplemented with 5.95 g/mL 4-(2-hydroxyethyl)-1-piperazineethanesulfonic acid (HEPES), always maintaining the percentage of water under 3% (v/v) in the sample. Heparin concentrations tested ranged from 200 to 0.09 μg/mL, and the DHP amount was accordingly adjusted considering the corresponding *w*_DHP_/*w*_heparin_ ratio previously established for each DHP. In addition, the highest water percentage added was assayed to check its effect on parasite growth, while nontreated cells were established as positive growth controls and 200 μg/mL heparin was employed as the negative growth control. Samples were added to the *P. falciparum* culture (ring stage), in triplicate, and incubated at 37 °C under hypoxia for 48 h. For flow cytometry analysis, pRBCs were diluted in PBS to a final concentration of 1–10 × 10^6^ cells/mL. To label the nuclei, 250 nM Syto-11 (Thermo Fisher Scientific) was included in the mixture. Samples were analyzed in an LSRFortessa 4 laser cytometer (BD Biosciences) with a high-throughput screening reader. Briefly, the cell population of interest, erythrocytes (both infected and non-infected), was selected by size (FSC, forward scatter) and complexity (SSC, side scatter); then, fluorescence was analyzed at λexcitation/emission = 488/525 nm. Flow rate was set at 1 μL/s, and 20,000 events per well were recorded. In addition, the parasitemia of randomly chosen wells was corroborated by Giemsa staining, followed by examination with an optical microscope (NIS Elements F 3.0, Nikon Instruments Inc., New York, US), using Plasmoscore 1.3 software (Burnet Institute, Melbourne, Australia) to facilitate counting.

### 2.7 *Ex Vivo* Production of Ookinetes and Targeting

To evaluate the ookinete targeting of DHP(G4)-MPA-Rh/hep, ookinetes were produced *ex vivo* following a previously reported protocol.^13,37^ Briefly, *Plasmodium berghei* CTRP-GFP parasites (which express green fluorescent protein when reaching ookinete stage; kindly provided by Dr. Inga Siden-Kiamos)^38^ were intraperitoneally (i.p.) administered to a BALB/c donor mouse (Janvier Laboratories, Le Genest-Saint-Isle, France). After 4 days, blood extracted from the donor mouse was used to infect i.p. a second mouse that was previously treated with phenylhydrazine (200 μL of a 6 mg/mL solution in PBS) to enhance reticulocyte production. Four days after infection was established in the second mouse, exflagellation events were checked by microscopic examination; if at least one event per field was not observed, blood collection could be delayed 1 more day. Blood carrying gametocytes was collected by intracardiac puncture and immediately diluted in 30 mL of ookinete medium (10.4 g/L RPMI supplemented with 2% w/v NaHCO_3_, 0.05% w/v hypoxantine, 0.02% w/v xanthurenic acid, 50 units/mL penicillin, 50 μg/mL streptomycin, 20% heat-inactivated FBS, and 25 mM HEPES, pH 7.4). The culture was then incubated for 24 h at 21 °C under orbital stirring (50 rpm) to allow ookinete conversion. Then, 0.5 mL of ookinete culture was incubated with 0.5 mg/mL of either DHP(G4)-MPA/hep or DHP(G4)-MPA-Rh/hep for 1 h at room temperature, and samples were washed three times with PBS and nuclei counterstained with 2 μg/mL Hoechst 33342. Images were acquired as described above with a Zeiss LSM880 confocal fluorescence microscope (Jena, Germany). Each experiment was repeated at least on three biological replicates.

### 2.8 Ethical Issues

The human blood used in this work was from voluntary donors and commercially obtained from the *Banc de Sang i Teixits* (www.bancsang.net; Barcelona, Spain). The use of human blood purchased from the blood bank has been approved by the Clinical Research Ethics Committee from the Hospital Clínic de Barcelona (www.clinicbarcelona.org/ceim), with register number HCB/2018/1223. Blood was not collected specifically for this research; the purchased units had been previously discarded for transfusion, usually because of an excess of blood relative to anticoagulant solution. Prior to their use, blood units underwent the analytical checks specified in the current legislation. Before being delivered to us, unit data were anonymized and irreversibly dissociated, and any identification tag or label had been removed in order to guarantee the nonidentification of the blood donor. No blood data were or will be supplied, in accordance with the current Spanish *Ley Orgánica de Protección de Datos* and *Ley de Investigación Biomédica*. The blood samples will not be used for studies other than those made explicit in this research.

For assays involving the use of mice, in the presence of toxic effects including, among others, >20% reduction in weight, aggressive and unexpected behavior, or the presence of blood in feces, animals were immediately anesthetized using a 100 mg/kg ketamine plus 10 mg/kg xylazine mixture and sacrificed by cervical dislocation. The animal care and use protocols followed adhered to the specific national and international guidelines in accordance with the current Catalan (D 214/1997/GC) and Spanish laws (RD 53/2013; order ECC/566/2015) and the corresponding European Directive (2010/63 EU). The animal procedures used in this work have been approved by the Animal Experimentation Commission from the Generalitat de Catalunya (“*Determinació de l’activitat antiplasmodial de diferents nanovectors en models murins de malària*”, Project 10100, Register numbers FUE-2018-00751856 and ID Y16ZTHXFR).

## 3 Results and Discussion

### 3.1 Formation of DHP–Heparin Complexes

**Figure 2:**
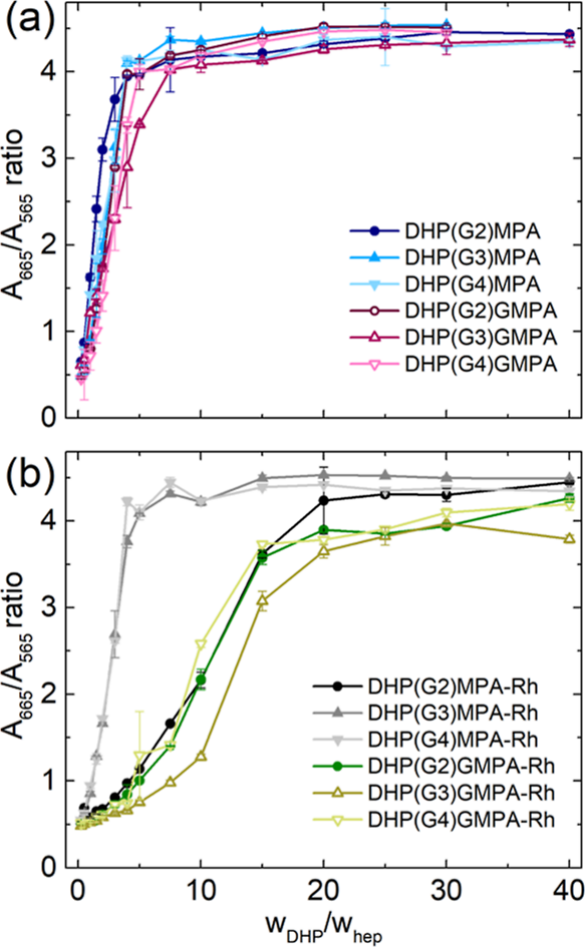
Determination of the complexation ratios between heparin and DHPs, either (a) without or (b) with Rh present on the DHP surface. Data are represented as mean ± SD (*n* = 2).

The representation of the intensity ratio between the absorbances (A_665_/A_565_) *versus* the weight ratio of both molecules involved in the complex formation (*w*_DHP_/*w*_heparin_) allowed to calculate the ratio at which heparin is completely complexed by the DHP, when the curve reached a plateau (Figure 2 and Table 1). Among the series of unlabeled DHPs (Figure 2a), little differences were found in their ability to complex heparin. Namely, all the DHPs of the *bis*-MPA series could totally complex heparin from a ratio 4:1 (*w*_DHP_/*w*_heparin_). Within the *bis*-GMPA series, a slight increase in the amount of DHP required to complex the heparin was observed, being the ratio 5:1 for the larger DHP, DHP(G4)-GMPA, and 7.5:1 for DHP(G3)-GMPA. Regarding the heparin complex formation with Rh-labeled DHPs (DHPs-Rh) (Figure 2b), it is relevant to indicate that Rh absorbance did not interfere with the measurements at the concentrations assayed. An increment in the *w*_DHP_/*w*_heparin_ ratios was observed in some cases compared to the DHPs without the fluorophore.

**Table 1:** Determined DHP/hep *w*/*w* Ratio for the Total Complexation of Heparin by DHPs and DHPs-Rh^a^

	*w*_DHP_/*w*_hep_	
	no Rh	with Rh
DHP(G2)-MPA	4	20
DHP(G3)-MPA	4	7.5
DHP(G4)-MPA	4	4
DHP(G2)-GMPA	4	15
DHP(G3)-GMPA	7.5	20
DHP(G4)-GMPA	5	15

a Data extracted from Figure 2.

This increment in the amount of DHP-Rh required to fully complex heparin could be due to steric hindrance imposed by Rh on the surface of the DHPs, which, although it is present in a small amount (less than 1% of the peripheral groups), might reduce the availability of amino groups for heparin interaction. Interestingly, among the DHP(Gn)-MPA-Rh series, an influence of the DHP core generation, and thus of the number of peripheral cationic groups, was observed in their ability to complex heparin (Figure 2b and Table 1). DHP(G3)-MPA-Rh and DHP(G4)-MPA-Rh fully complexed heparin at a low ratio, while the DHP with less terminal amino groups, DHP(G2)-MPA-Rh, required a higher amount of dendritic material to displace MB and completely complex heparin. Regarding the DHP(Gn)-GMPA-Rh series, this influence of the number of peripheral groups was also observed, although to a lower extent, with *w*_DHP_/*w*_heparin_ ratios varying from 15:1 to 20:1.

**Figure 3:**
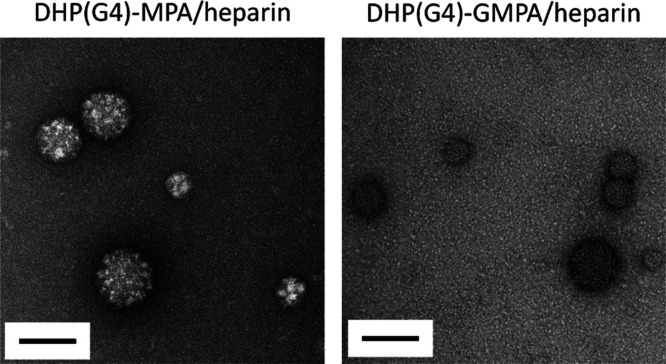
TEM images of the complexes formed by DHPs(G4) and heparin. Scale bars: 100 nm.

The morphology of the complexes formed at the established ratios with heparin by the largest DHPs from the two series, namely, DHP(G4)-MPA and DHP(G4)-GMPA, was studied by TEM (Figure 3). Whereas free DHPs formed unimolecular micelles,^33^ the complexes with heparin resulted in larger spherical structures likely due to heparin acting as a micelle crosslinker. Nanospheres with a diameter between 40 and 100 nm were observed for DHP(G4)-MPA/hep and from 30 to 80 nm for DHP(G4)-GMPA/hep. The smaller size observed in the nanospheres of the GMPA complex compared to the MPA derivative could be caused by the presence of inner amide groups in the structure of the former, which could also establish hydrogen bonds with heparin and yield more compact structures.

### 3.2 *In Vitro* Cytotoxicity Assays

**Figure 4:**
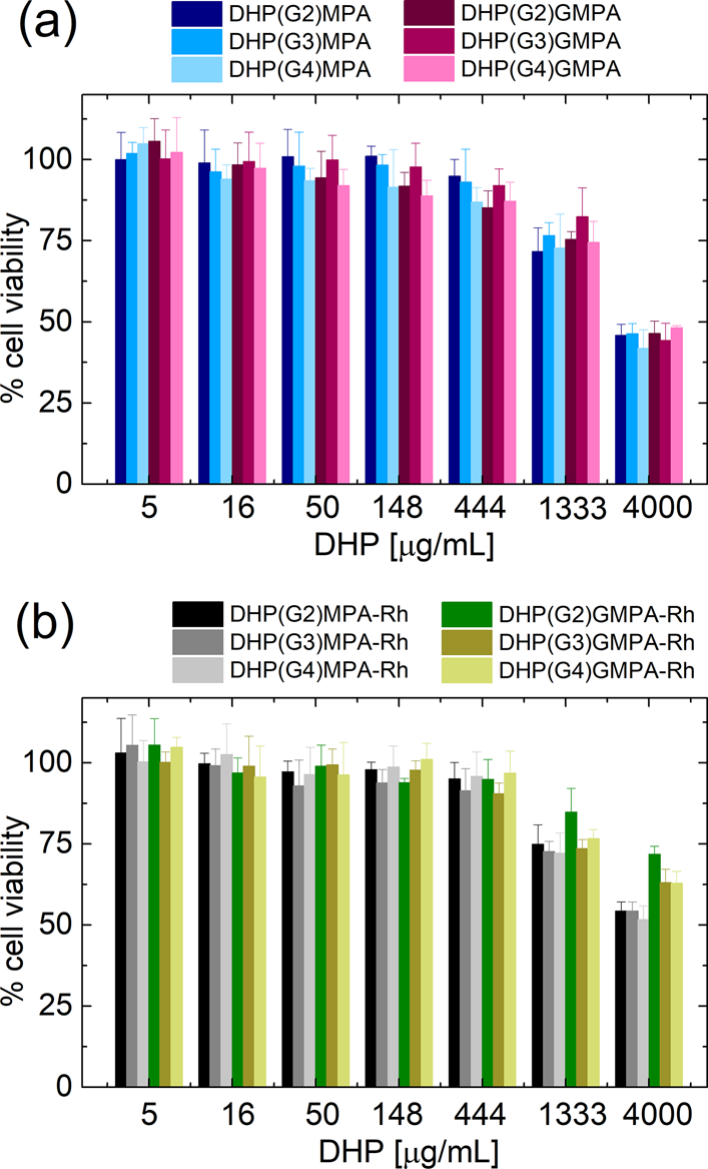
Cytotoxicity assayed in HUVECs, relative to an untreated control, of the series of (a) DHPs and (b) DHPs with Rh. Data are represented as mean ± SD (*n* = 3). Statistical analysis data are provided in the Supporting Information (Table S1).

Before assessing antimalarial activity, the unspecific cytotoxicity of the unlabeled and labeled DHPs was investigated in HUVECs. All the compounds assayed were found to have low cytotoxicity up to 444 μg/mL, with viability levels above 80%, and moderate cytotoxicity up to 1333 μg/mL, where cell viability remained above 70% (Figure 4).

At the highest concentration assayed, 4 mg/mL, DHPs exhibited significant cytotoxicity with viability levels between 40 and 50%. As observed in Table S1 (Supporting Information), all DHPs present cytotoxicity values statistically different between 1333 and 4000 μg/mL, except DHP(G2)-GMPA labeled with rhodamine. Interestingly, DHPs-Rh showed a lower cytotoxicity at that concentration, with viability values ranging from 50 to 70%. It can be speculated that the lower amount of free amino groups in the compounds that bear Rh on the surface contributes to a lower cytotoxic effect. It must be noted that the DHPs labeled with Rh of the *bis*-MPA series had a slightly increased cytotoxic effect relative to those of the *bis*-GMPA series. This observation is also in agreement with the possible reduction of amino terminal groups exposed on the outer part of the DHP in the case of the *bis*-GMPA series, probably due to intramolecular bonds favored by the inner amides present in them.

### 3.3 Targeting to pRBCs and Ookinetes

**Figure 5:**
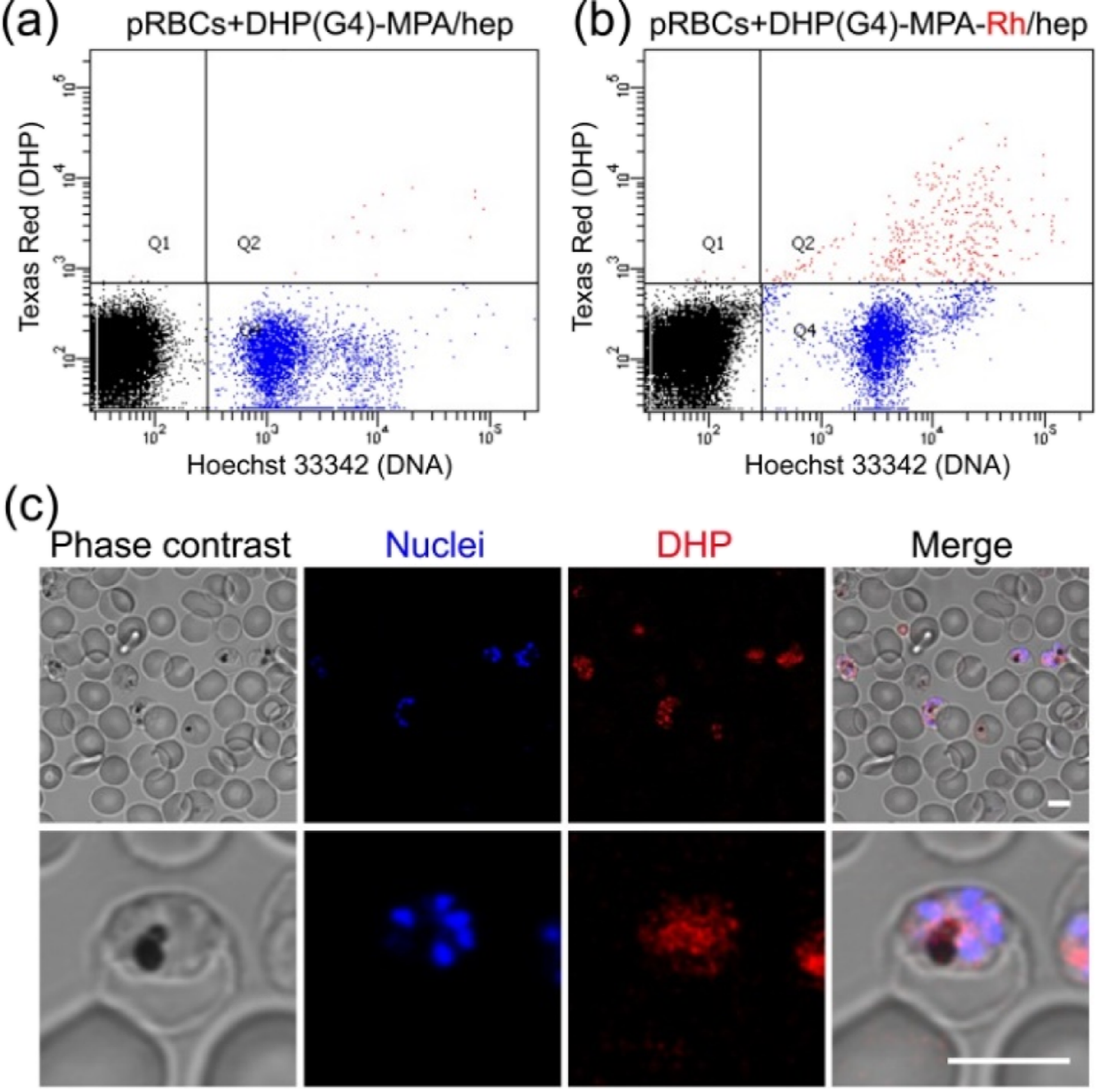
Targeting of DHP(G4)-MPA-Rh/hep to pRBCs. An *in vitroP. falciparum* culture was incubated with 0.5 mg/mL DHP(G4)-MPA-Rh/hep and analyzed by (a,b) flow cytometry and (c) confocal fluorescence microscopy. (a) Unlabeled DHP control. Scale bar: 5 μm.

The specific binding of the complexes to parasitized RBCs and ookinetes was tested in *in vitroP. falciparum* cultures. Based on the good targeting results provided by our previously reported DHP,^25^ we decided to perform these studies with DHP(G4)-MPA-Rh/hep as it resembles the structure of the former. Namely, both contain *bis*-MPA-based dendrons and a similar amount of terminal ammonium groups in their structure. In addition, DHP(G4)-MPA-Rh presents a low DHP/heparin ratio (see Table 1), which is convenient to reduce the amount of required DHP and hence minimize the cytotoxicity associated to large concentrations (see Figure 4). Using pRBCs synchronized at late blood stages, targeting of the Rh-labeled DHP toward pRBCs was explored by flow cytometry (Figure 5b). Although the fraction of targeted pRBCs was low (10.8%), the displacement observed for the entire cell population suggested the presence of aggregation events affecting the assay’s readout.

On the other hand, confocal fluorescence microscopy analysis (Figure 5c) indicated specific targeting of DHPs to pRBCs *versus* nonparasitized RBCs (90 and <1% targeting, respectively), in accordance with existing data that showed a similar specific binding exhibited by heparin to *Plasmodium* blood stages.^7,8^ Regarding eventual clinical applications, the nanocarrier concentration in blood will need to be carefully considered in order to prevent nanoparticle aggregation and/or RBC agglutination. At the concentration used in flow cytometry assays (2.5 mg/mL), all DHPs exhibited significant toxicity (Figure 4). Concentrations below 0.5 mg/mL, showing cell viability levels above 80%, will be more adequate for future *in vivo* assays.

Previous results had also reported binding of fluorescein-labeled heparin to the ookinete, the motile *Plasmodium* zygote present in the mosquito midgut,^12^ and a heparin-mediated inhibition of ookinete development.^13^ The targeting of mosquito stages of *Plasmodium* offers some clear a priori advantages,^14^ such as the avoidance of clinical assays that significantly increase the cost of therapies, an undesirable scenario for antimalarials that have to be used in low- and medium-income countries. We therefore explored if DHP(G4)-MPA-Rh/hep would also target this stage of the parasite. Our results indicated an absence of ookinete targeting (Figure S2), which could be due to conformational rigidity or steric hindrance imposed by DHP(G4)-MPA-Rh binding to heparin, preventing its interactions with receptors on the ookinete surface.

### 3.4 *In Vitro* Antimalarial Activity of DHP–Heparin Complexes

**Figure 6:**
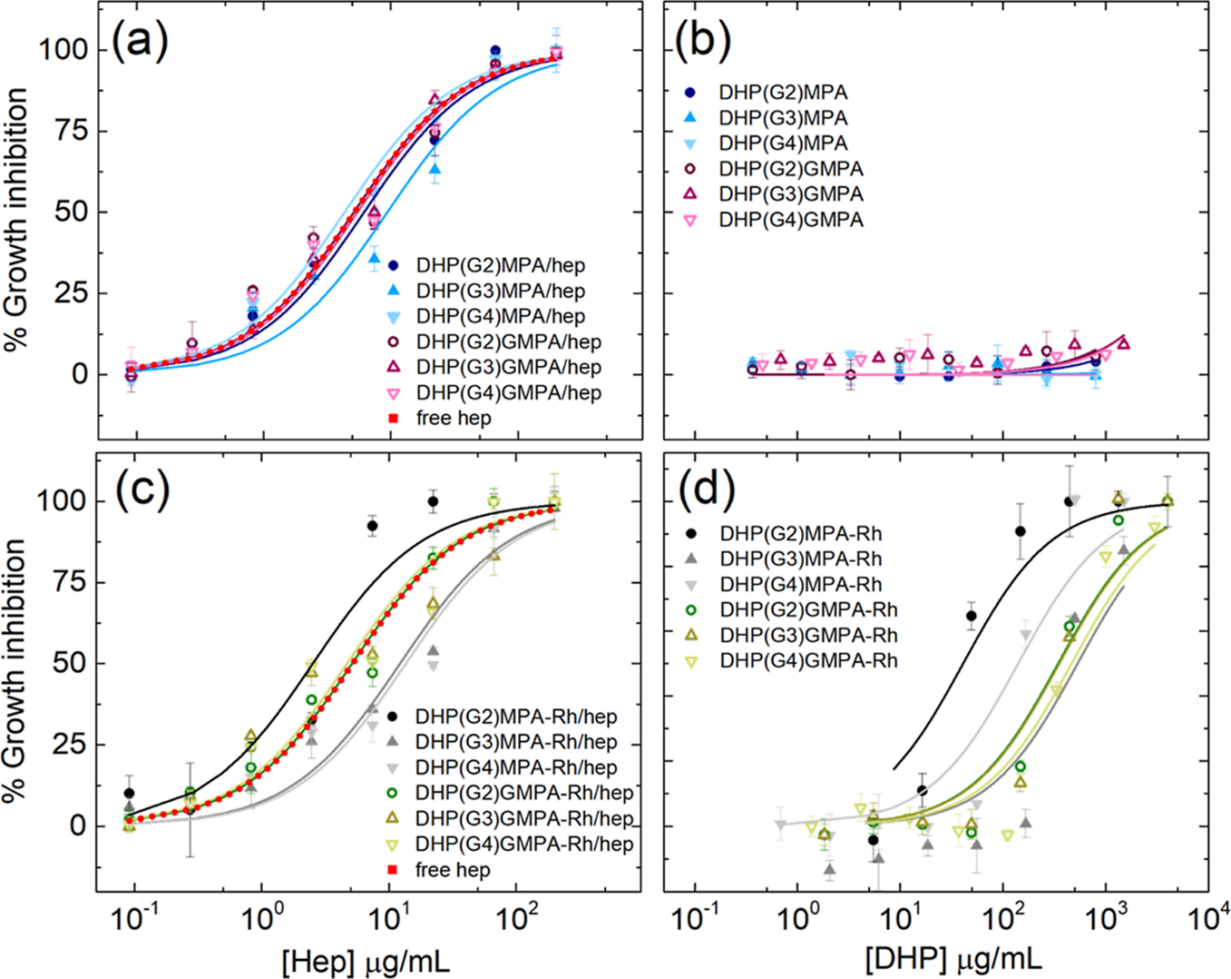
*P. falciparum* growth inhibition assays of (a) DHPs/hep complexes, (b) free DHPs, (c) DHPs/hep complexes labeled with rhodamine, and (d) free DHPs labeled with rhodamine. Free heparin appears as the control in (a,c). Data are represented as mean ± SD (*n* = 3).

The complexes formed by unlabeled DHPs and heparin (Figure 6a) presented antiparasitic activities close to that of free heparin, which had an IC50 of *ca.* 5 μg/mL (∼400 nM) (Table 2). Specifically, all the DHPs of the *bis*-GMPA series in combination with heparin presented similar IC50 values, ranging from 5.1 to 5.4 μg/mL (Table 2), whereas within the *bis*-MPA series, more variability was found. In the case of the largest compound in combination with heparin, DHP(G4)-MPA/hep, its IC50 (4.3 μg/mL) indicated a slight improvement of inhibitory activity relative to free heparin, while the other two complexes, DHP(G2)-MPA/hep and DHP(G3)-MPA/hep, did not show this effect. In addition, the *Plasmodium* growth inhibition activity of the free DHPs was assayed and it was observed that they did not have significant antiparasitic activity by themselves (Figure 6b and Table 2), confirming that the activity previously observed for the complexes with heparin was due to the appropriate complexation of heparin on the DHP structures. The series of DHPs labeled with Rh was also tested in *in vitroP. falciparum* cultures (Figure 6c,d). The low concentration of Rh present in the samples did not impair the measurement of Syto-11 fluorescence at the selected wavelength. In this case, little variation was found again within the activity of the complexes of the *bis*-GMPA series, while more divergent results were found for the *bis*-MPA-containing complexes. However, the activity of free DHPs-Rh revealed a certain inhibitory activity inherent to the labeled DHPs themselves (Figure 6d), and thus, the interpretation of the results must consider the amount of DHP-Rh required to form the complex with heparin.

**Table 2:** IC50 Values in *P. falciparumIn Vitro* Cultures for All the Combinations Tested

	free DHPs		DHP–heparin complexes	
	IC50 (μg/mL)		IC50 (μg/mL; μM)	
compound	unlabeled	+Rh tag	unlabeled	+Rh tag
free heparin	-	-	5.2 ± 1.0, 0.40 ± 0.04	-
DHP(G2)-MPA	>1000	40.9 ± 13.8	6.2 ± 1.0, 0.47 ± 0.04	2.5 ± 0.7, 0.19 ± 0.03
DHP(G3)-MPA	>1000	531.7 ± 224.0	9.4 ± 2.1, 0.72 ± 0.09	12.2 ± 2.4, 0.94 ± 0.11
DHP(G4)-MPA	>1000	140.3 ± 48.0	4.3 ± 0.6, 0.33 ± 0.02	13.5 ± 3.2, 1.04 ± 0.14
DHP(G2)-GMPA	>1000	344.2 ± 85.7	5.1 ± 1.1, 0.39 ± 0.05	5.1 ± 0.9, 0.39 ± 0.04
DHP(G3)-GMPA	>1000	352.4 ± 104.6	5.4 ± 0.6, 0.42 ± 0.03	4.7 ± 1.2, 0.36 ± 0.06
DHP(G4)-GMPA	>1000	464.8 ± 129.6	5.3 ± 1.0, 0.41 ± 0.04	4.7 ± 1.2, 0.36 ± 0.05

The antimalarial potential of Rh has been previously reported.^39,40^ In this regard, the combination that showed a better *Plasmodium* inhibitory performance with an IC50 of 2.5 μg/mL, DHP(G2)-MPA-Rh/hep, required a *w*_DHP_/*w*_heparin_ ratio 20:1 to establish the complexes, which led to a larger amount of Rh in the sample than in the cases of DHP(G3)-MPA-Rh/hep or DHP(G4)-MPA-Rh/hep, whose respective complexation ratios were 7.5:1 and 4:1 and their IC50 was higher than 12 μg/mL. Surprisingly, the antiparasitic activities of DHP(Gn)-GMPA-Rh complexes with heparin (IC50 between 4.7 and 5.1 μg/mL) were slightly better but did not differ too much from those of the homologues without the fluorophore (Figure 6a), even though the ratios of complexation (*w*_DHP_/*w*_heparin_ 15:1–20:1) indicated that a high amount of Rh was present in the complex. The presence of Rh should affect minimally the antimalarial activity of heparin-containing DHPs since, at the ratio selected here for the labeling, Rh has little effect on parasite viability relative to the comparatively much higher antiplasmodial activity of the heparin concentration used. Although this is so for DHP(Gn)-GMPA/hep complexes, the marked activity improvement for DHP(G2)-MPA/hep (IC50 drop from 0.47 to 0.19 μM) and decrease for DHP(G3)- and DHP(G4)-MPA/hep formulations (IC50 increases from 0.72 and 0.33 to 0.94 and 1.04 μM, respectively) were somewhat unexpected. As suggested above to explain the lack of ookinete targeting, conformational modulations imposed on heparin by its interactions with the different architectures of DHPs might be responsible for the observed variations on its effect on parasite survival when conjugated to DHPs of the MPA series.

The malaria parasite has been relentlessly evolving resistance to every single drug used against it for the last 100 years,^41^ which has rendered once promising compounds, such as the quinine-derived quinolines, virtually useless today in many endemic regions. Projects for the discovery of new antimalarials have brought to the market artemisinin combination therapies (ACTs), but by 2016, the emergence of resistance in *P. falciparum* to artemisinin and most ACT partner drugs was detected in the Greater Mekong Subregion,^42^ and recently, the independent evolution of artemisinin resistance has also been reported in Africa^43^ and South America.^44^ This situation is worsened by the predicted climate change-driven regional expansion of the parasite and the chronic insufficient funding for malaria research. This alarming scenario calls for the urgent development of new drugs of easy and cost-affordable production, with little-exploited targets in the malaria parasite, having several molecular targets in the pathogen and acting through new mechanisms of action not shared by currently used drugs. New promising compounds, such as the recently discovered YAT2150,^45^ might require the availability of nanocarriers such as the heparin-targeted DHPs characterized here in order to improve pharmacokinetic profiles and for targeted delivery to *Plasmodium*-infected cells. Such specific targeting will be essential to limit the emergence of resistance in the pathogen to future drugs because the administered doses will result in higher local concentrations reaching the parasite and therefore in a reduction of the likelihood of resistance evolution.

Current ACTs require that one drug (usually with potent antiplasmodial activity like artemisinin or a derivative of it) must be immediately available to act rapidly to alleviate the acute clinical symptoms, while a second partner drug, usually with a lower activity but with a much longer blood residence time, will kill off late-developing parasites. This ACT design must be taken into account for future nanocarrier-based targeted delivery strategies by engineering the drug-encapsulating structures in such a way that the fast-acting drug is rapidly released while the slow-acting compound will be leaking from the nanovector at a much slower pace. However, future combination therapies might involve drugs that need to be released together, for example, because they are both highly potent and have different targets in the parasite. In such cases, the sequential release required for ACTs would not be required.

## 4 Conclusions

Here, we show that hyperbranched polyester polymers of G2, G3, and G4 functionalized with *bis*-MPA or *bis*-GMPA dendrons result into dendronized hyperbranched polymers that present good properties as heparin nanocarriers. Regarding the complexation of heparin, no effect of core generation or of the type of dendron has been observed in the case of unlabeled DHPs, but some notable differences are appreciated in the case of Rh-labeled compounds. Indeed, the presence of the labeling agent significantly increases the DHP–heparin complexation ratios in all DHPs, except for DHP(G4)-MPA-Rh. These DHP–heparin complexes have shown low unspecific cytotoxicity and selective targeting to *Plasmodium*-infected red blood cells *versus* noninfected erythrocytes. All the DHP–heparin complexes efficiently maintain the inhibition effect of heparin on the growth of *P. falciparumin vitro* cultures. Interestingly, whereas unlabeled DHPs do not significantly affect the growth of *Plasmodium*, the presence of Rh provides certain inhibitory activity. Comparing both series in terms of antiplasmodial activity, all labeled and unlabeled *bis*-GMPA derivatives effectively maintain the inhibitory capacity of free heparin, with similar and even slightly lower IC50 values, while the *bis*-MPA derivatives show higher variability with a marked improvement for the biggest unlabeled derivative DHP(G4)-MPA/hep. Indeed, by considering together heparin complexation efficacy, cell viability, and *P. falciparum* growth inhibition potency, unlabeled DHP(G4)-MPA exhibits the best performances as a heparin nanocarrier for antimalarial purposes among all presented DHP derivatives. The dual activity of heparin as a targeting element of nanocarriers capable of transporting drugs and as an antimalarial element by itself presents heparin-coated DHPs as potential interesting components of future antimalarial targeted drug delivery strategies.

## Supporting Information Available

UV–visible spectra for the MB competition assay, confocal images for the targeting of ookinetes, and heparin release studies, as well as statistical analysis of cytotoxicity data (PDF)
