# Supplementary material for: Heparin-Coated Dendronized Hyperbranched Polymers for Antimalarial Targeted Delivery
Source: ACS Appl Polym Mater. 2022 Dec 30;5(1):381–90. doi: 10.1021/acsapm.2c01553 (PMC9844211; doi:10.1021/acsapm.2c01553)
Supplement: Supplementary file 1 — ap2c01553_si_001.pdf [file ap2c01553_si_001.pdf]

Supporting information

Heparin-coated dendronized hyperbranched polymers for antimalarial targeted delivery

María San Anselmo,[1] Elena Lantero,[2,3,4] Yunuen Avalos-Padilla,[2,3,4] Inés Bouzón-Arnáiz,[2,3,4] Miriam Ramírez,[2,3,4] Alejandro Postigo, [1] José Luis Serrano,[1] Teresa Sierra,[1] Silvia Hernández-Ainsa,[1,5]* Xavier Fernàndez-Busquets[2,3,4]**

1. Instituto de Nanociencia y Materiales de Aragón (INMA), Departamento de Química Orgánica-Facultad de Ciencias, CSIC-Universidad de Zaragoza, 50009, Spain.

2. Nanomalaria Group, Institute for Bioengineering of Catalonia (IBEC), The Barcelona Institute of Science and Technology, Baldiri Reixac 10-12, 08028 Barcelona, Spain.

3. Barcelona Institute for Global Health (ISGlobal, Hospital Clínic-Universitat de Barcelona), Rosselló 149-153, 08036 Barcelona, Spain.

4. Nanoscience and Nanotechnology Institute (IN2UB), University of Barcelona, Martí I Franquès 1, 08028 Barcelona, Spain.

5. ARAID Foundation, Government of Aragón, Zaragoza, 50018, Spain.

Section S1. Heparin complexation

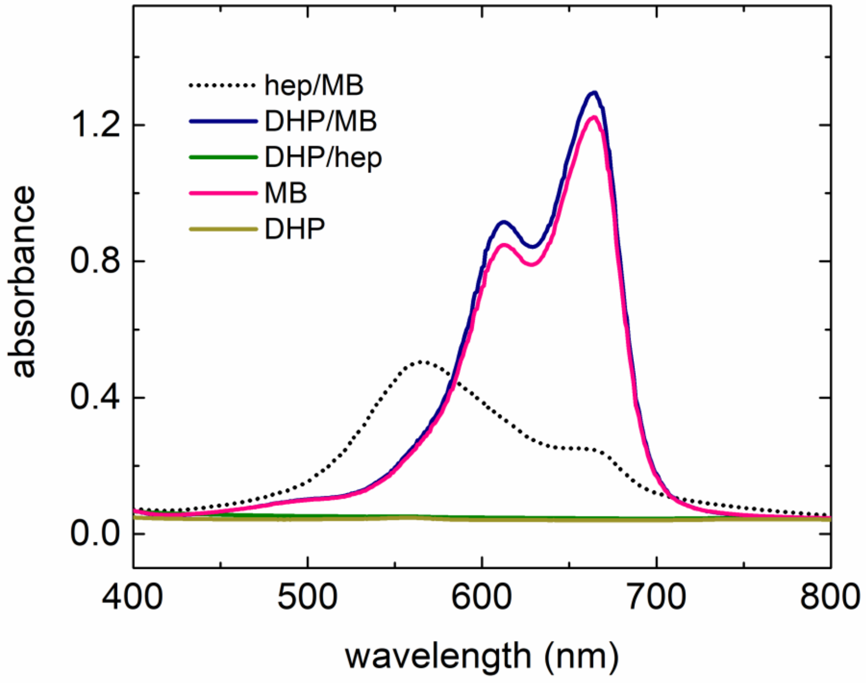

Figure S1. UV-visible spectra identification of the maximum wavelength in each combination of the MB competition assay. Samples were measured in 10 mM tris-HCl (pH 7.6) at 50 µM (MB), 100 µg/mL (DHP) and 10 µg/mL (heparin).

Section S2. Targeting to ookinetes

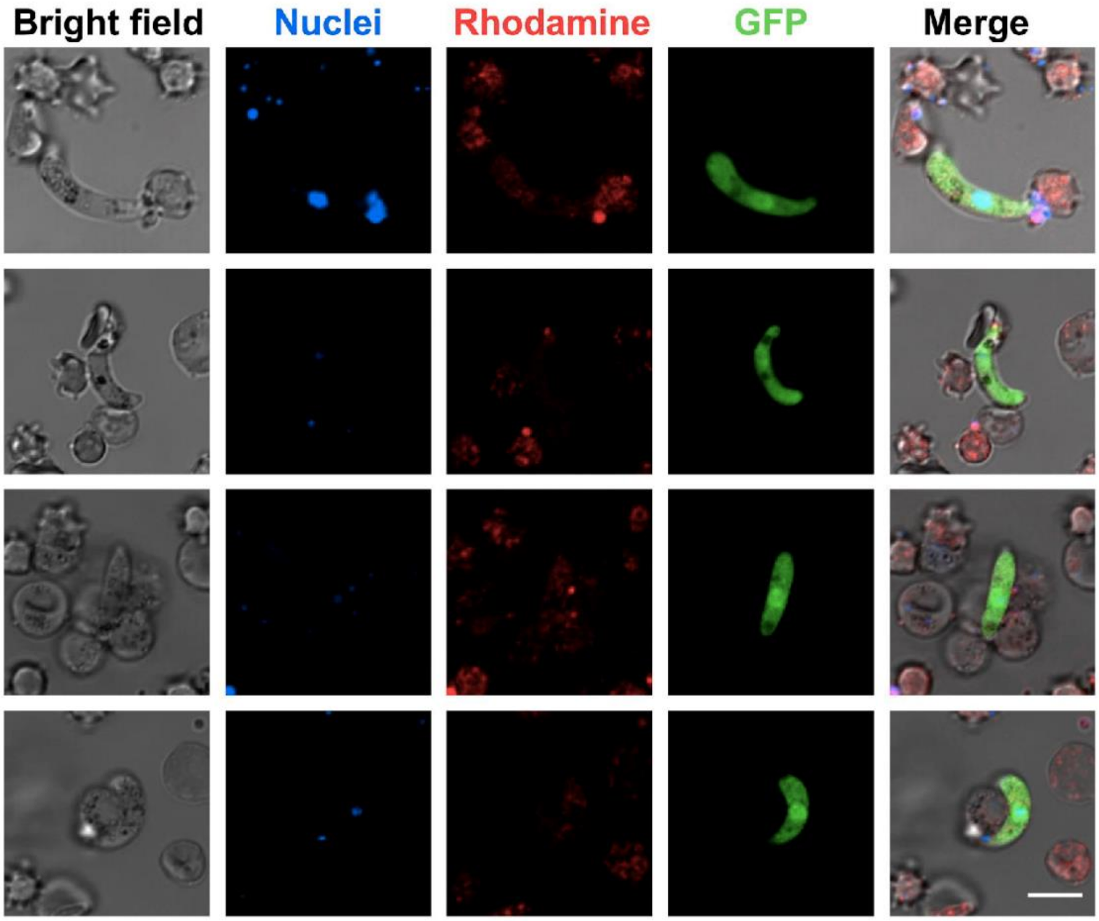

Figure S2. Targeting of DHP(G4)-MPA-Rh/hep to ookinetes. Representative images of *ex vivo* produced ookinetes incubated with 0.5 mg/mL of DHP(G4)-MPA-Rh/hep and analyzed by confocal fluorescence microscopy. Scale bar: 5 µm.

Section S3. Heparin release study

Heparin release from DHPs was evaluated by incubating the nanoformulations in RPMI media at 37 ºC for different time periods up to 24 hours. All DHPs were loaded to contain 20 µg of heparin. After incubation, samples were loaded in a non-denaturing polyacrylamide gel (6% in 20 mM tris-HCl buffer, pH 8.8) and run at 100 V for 40 min. Then gels were stained with Alcian blue (Iduron Ltd.) following the supplier recommendations. Released heparin can be observed in the gel (Fig S3a) and the quantification of the intensity of the bands at each time point with ImageJ allowed to estimate the percentage of heparin released versus time (Figure S3b). Although both complexes show similar kinetic profiles, a slightly faster release is observed for the MPA-derived DHP. A retarded release is observed for this DHP when it is incubated in RPMI supplemented with fetal calf serum (10% FCS in RPMI) (Fig S3c). In previous reports we have investigated the biodegradability of dendrons based on bis-MPA (J. Movellan et al. *Biomaterials* 2014, 35:7940-7950) and bis-GMPA (A. Lancelot et al. *J. Mater. Chem. B* 2018, 6:3956-3968). In both cases, at the temperature (37 ºC) and pH (7.4) of *P. falciparum* growth inhibition assays, the ester cleavage of the glycine units from the dendritic block begins after *ca.* 1 h of incubation, thus favoring heparin release in the present case. The hydrolytic degradation of the polyester and poly(esteramide) skeletons is accelerated in acidic conditions. We can therefore assume that our current DHPs may follow similar degradation patterns.

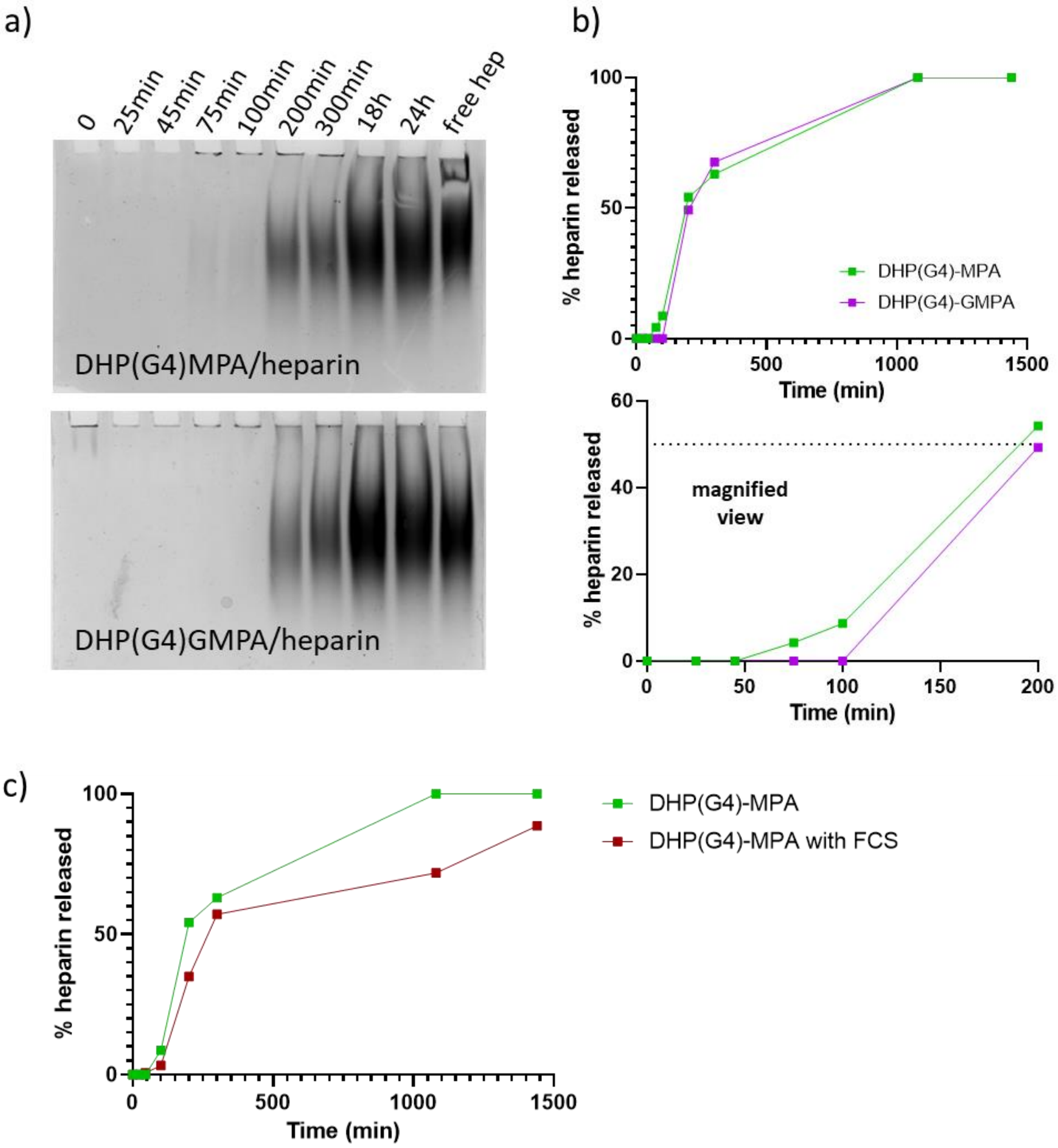

Figure S3. Heparin release study from DHP(G4)-MPA/heparin and DHP(G4)-GMPA/heparin complexes. a) Polyacrylamide gel electrophoresis analysis of heparin release after different times of incubation in RPMI at 37 ºC; b) graphical representations of the percentage of heparin released at each time point from DHP(G4)-MPA/heparin (green) and DHP(G4)-GMPA/heparin (purple) incubated in RPMI at 37 ºC; c) graphical representation of the percentage of heparin released from DHP(G4)-MPA/heparin incubated for different times in RPMI (green) or in RPMI supplemented with 10% FCS (red).

Section S4. Statistical analysis of cytotoxicity data

Table S1 collects the *p* values obtained by the statistical analysis of the data in Figure 4 and it was performed using GraphPad Prism (GraphPad Inc.). Cell viability data of the same compound at 1333 µg/mL and 4000 µg/mL were compared using unpaired two tailed t -test. *p* values of <0.05 were considered significant. ns: non-significant.

	**Analysed concentrations (µg/mL)**		**Analysed concentrations (µg/mL)**
**Free DHPs**	**1333 *vs* 4000**	**DHP labelled with Rh**	**1333 *vs* 4000**
DHP(G2)-MPA	0.0133	DHP(G2)-MPA	0.0141
DHP(G3)-MPA	0.0006	DHP(G3)-MPA	0.0018
DHP(G4)-MPA	0.0198	DHP(G4)-MPA	0.0127
DHP(G2)-GMPA	0.001	DHP(G2)-GMPA	0.0777, ns
DHP(G3)-GMPA	0.0061	DHP(G3)-GMPA	0.0272
DHP(G4)-GMPA	0.0193	DHP(G4)-GMPA	0.008
